# Healthy Ageing: A Decision-Support Algorithm for the Patient-Specific Assignment of ICT Devices and Services

**DOI:** 10.3390/s23041836

**Published:** 2023-02-07

**Authors:** Agnese Brunzini, Manila Caragiuli, Chiara Massera, Marco Mandolini

**Affiliations:** Department of Industrial Engineering and Mathematical Sciences, Università Politecnica delle Marche, Via Brecce Bianche 12, 60131 Ancona, Italy

**Keywords:** personalised care, elderly, decision-support algorithm, healthy ageing, wearable sensors, digital health

## Abstract

In response to rapid population ageing, digital technology represents the greatest resource in supporting the implementation of active and healthy ageing principles at clinical and service levels. However, digital information platforms that deliver coordinated health and social care services for older people to cover their needs comprehensively and adequately are still not widespread. The present work is part of a project that focuses on creating a new personalised healthcare and social assistance model to enhance older people’s quality of life. This model aims to prevent acute events to favour the elderly staying healthy in their own home while reducing hospitalisations. In this context, the prompt identification of criticalities and vulnerabilities through ICT devices and services is crucial. According to the human-centred care vision, this paper proposes a decision-support algorithm for the automatic and patient-specific assignment of tailored sets of devices and local services based on adults’ health and social needs. This decision-support tool, which uses a tree-like model, contains conditional control statements. Using sequences of binary divisions drives the assignation of products and services to each user. Based on many predictive factors of frailty, the algorithm aims to be efficient and time-effective. This goal is achieved by adequately combining specific features, thresholds, and constraints related to the ICT devices and patients’ characteristics. The validation was carried out on 50 participants. To test the algorithm, its output was compared to clinicians’ decisions during the multidimensional evaluation. The algorithm reported a high sensitivity (96% for fall monitoring and 93% for cardiac tracking) and a lower specificity (60% for fall monitoring and 27% for cardiac monitoring). Results highlight the preventive and protective behaviour of the algorithm.

## 1. Introduction

Throughout the world, every country is experiencing the population ageing phenomenon. This global transformation mainly concerns two counterposed effects: a significant increase in life expectancy and a rapid decrease in birth rate. Consequently, considerable changes in the age distribution of societies will occur. People aged 65 years and over are expected to live an additional 17–19 years from 2015 to 2050. The world will have a higher number of older adults than children. Elders will grow from 9% in 2019 to 16% in 2050 [1].

Although longevity is undoubtedly one of the most remarkable successes in human history, physiological changes, including the loss of both intrinsic capacity and functional ability and the onset of chronic diseases, may occur with increasing age. Therefore, this demographic transformation will create challenges within the health and social care systems, causing economic and social implications. However, it will also allow modern societies to explore new healthy and active ageing strategies [2].

Information and Communication Technology (ICT) may be crucial for implementing such an innovative approach, being the key enablers of digital tools supporting the health and social care sectors. Indeed, they can promote person-centred care and prevent or delay frailty conditions by improving quality of life, health, and well-being. Several ICT solutions and services have experimented with various settings. Some of these paradigms positively impact eldercare [3]. Specifically, amplified by the recent COVID-19 pandemic, digital platforms have gained interest in the remote monitoring of older patients [4]. Some research studies carried out on this emerging trend have focused on the management of specific aspects, symptoms, or diseases within the frailty framework, such as balance and risk of falling [5], psychological and cognitive support [6], measurement of physiological parameters (i.e., blood pressure, weight, and heart rate) [7,8], abnormal activity detection [9], and medication support [10]. However, the literature lacks digital information platforms able to deliver coordinated health and social care services for older people to cover their needs comprehensively. Indeed, although some works that start moving in this direction exist [11,12], the integration of clinical and social aspects is still inadequate.

The present work is part of the Italian National Project called “*MOSAICO—Models, products, and services to make the life of frailty people socially active and inclusive in communities spread across the territory*”. MOSAICO is an initiative that has received funding from the Ministry of Enterprises and Made in Italy (ex Ministry of Economic Development) and Marche Region under the grant agreement for innovation. The project aims to identify a new concept of assistance model and develop an innovative software platform (MOSAICO) to facilitate the design, management, and supervision of health and social services for the elderly. The MOSAICO system aims to enhance the quality of life of older people living in remote and low-density urbanisation areas of Central Italy, thanks to the adoption of personalised paths. The main objective is to prevent acute events, favour the elderly staying healthy at home while reducing hospitalisations, and make them active and independent in their countries. The second, but not less important objective, is to contrast the increasing depopulation phenomenon in the areas damaged by the earthquake in 2016. Those areas will be revitalised with improved and more accessible services.

For this purpose, local organisations and cooperatives are essential resources to be included in the project. They build a solid community-based network to support older people by addressing their specific needs. To meet the challenges posed by the ageing of the population, the project employs solutions entailing ICT that is driving the current digital transformation of health and well-being (namely Healthcare 4.0). Mobile and wearable devices embedded with sensors have a fundamental role in this new health monitoring concept [13], enhancing patient-centric, personalised, and proactive measures. These innovative systems dedicated to active ageing will enable older people to be safer, contributing to their staying independent and having an active social life. In this way, the negative impact of adverse events such as falls and cardiovascular diseases will be reduced.

The core of this interconnected digital framework for ageing is the MOSAICO platform. It is used (among several features) to collect patients’ clinical and social information acquired during the multidimensional screening phase of the project and from the ICT devices during the subsequent telemonitoring phase. The first phase consists of a wide-range assessment of the elderly, including motor-functional, cardiological, nutritional, psychological, and social domains. In the second phase, the patient is provided with a personalised set of products and services based on their characteristics and requirements. This software platform is not intended as a telemedicine system. However, it was designed to eventually interact with and complement one to extract relevant data while monitoring and self-monitoring the patient’s condition [14].

The purpose of the work and its significance lie in the counteraction against the frailty condition of elders. Crucial is the prompt identification of criticalities and vulnerabilities and the promotion of active and healthy habits. This approach to preventing or at least delaying frailty progression fits into a more comprehensive framework that desires to positively impact the traditional care model of the National Health System [15]. The goal is to move from therapeutic to proactive measures.

In this context, the present work aims to define and implement a decision-support algorithm that automatically assigns devices and services tailored to seniors’ clinical and social needs. Platform functionalities must be improved by including a logical method to identify the best health and social services model. This essential joining link between patient’s information and interventions would help professionals analyse the specific fragilities and contribute to carrying out a personalised path for elders. Importantly, technological solutions considered by the algorithm to improve self-sufficiency and awareness make it stand out among other studies focusing on integrated care programmes for the elderly [16]. Thus, the conception of the idea and its novelty come from the all-inclusive and personalised response the algorithm provides for older people’s needs. A higher number of predictive factors of frailty are considered to provide value to the single specificities of elders. Ease of use and compiling are two strong points of the algorithm to make it a suitable tool for every professional.

The proposed algorithm, implemented on the MOSAICO software platform, will make the product–service assignment more efficient and time-effective, thanks to the proper combination of specific features, thresholds, and constraints related to the ICT devices and patients’ characteristics. The adoption of a holistic approach is an indispensable condition for outlining a comprehensive profile of the patient. Conversely, the large amount of generated data may be challenging to manage; using an automatic decision-support tool becomes fundamental for proper patient-specific assignment.

The case study was carried out on a target population of 50 participants recruited from the MOSAICO project. Data involved a combination of self-reported, sensor-, and questionnaire-based information from the multidimensional sessions of the screening to explore the multifactorial nature of the frailty condition. These input data were used to feed a decision tree algorithm designed to match the specific characteristics of the patient’s profile and their inclination to the technology, as well as the technical features of the devices, and to generate as output the best set of devices and social services for that patient. This combined care plan established by the algorithm aspires to detect early markers of physical and mental declines in older people and to reverse or delay them through appropriate monitoring and care. Validation results are promising and show high sensitivity in patient-specific matching.

## 2. Materials and Methods

The present work aims to study and develop a decision-support algorithm to help clinicians find the best set of devices and services for older people based on their social and clinical needs. A personalised approach is fundamental to understanding the heterogeneous nature of the elderly population, which is characterised by high variability and numerous specificities and is influenced by complex factors, both intrinsic and extrinsic to the individual. 

**Figure 1:**
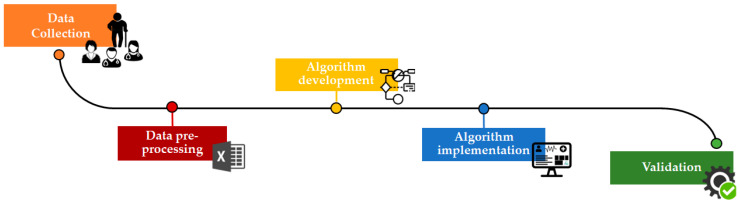
Workflow representing the steps of the developed methodology.

The proposed methodology to develop the decision-support model is shown in Figure 1. The principal points are the following:

- Multidimensional assessment: elders’ health evaluation by a multidisciplinary staff of clinical and social operators. The multidimensional screening of elders’ health allows for gathering the information representing the knowledge base of the reasoning system to determine a course of action.
- Data preprocessing: this is a crucial phase to make the data available for algorithm development. Data are cleaned and coded in a standardised format according to the nature of the acquired variables.
- Algorithm development: this is a crucial step in the developed methodology since it refers to the logic behind the decision-support tool. It follows a decision tree approach showing the various outcomes from a series of decisions. The identification of products and services is based on the patient’s profile.
- Algorithm implementation: it describes the process of converting the decisional model into code. Visual Basic for Applications (VBA) was chosen as the programming language for its ease of use, which is indispensable in a preliminary study.
- Validation: evaluation of the reliability and consistency of the algorithm itself through a specific validation protocol applied to a sample of participants.

In Section 2.1, the description of the data collection procedure is reported in detail. Section 2.2, Section 2.3 and Section 2.4 are devoted to the core steps of the methodology related to data cleaning, algorithm development, and implementation. Next, Section 2.5 focuses on the validation procedure. Section 3 reports the results and discussion.

### 2.1. Data Collection

#### 2.1.1. Multidimensional Assessment

This section highlights the variables assumed as input to the developed matching algorithm.

As a normal ageing process, the integrity and functionality of physiological systems reduce over time. The higher the biological age, the higher the probability of experiencing an adverse event such as falls or cardiovascular (CV) problems. Beyond non-modifiable risk factors (e.g., age, gender, race, or familiarity), the presence of comorbidities (e.g., hypertension, obesity, respiratory disease, diabetes, joint disease, sensory impairment, and mental health problems), and polypharmacy, there are additional factors that can be modified to prevent such adverse events. Behavioural factors include lifestyle aspects that can improve life quality and reduce morbidity and mortality among older adults. Adequate physical activity, healthy nutritional habits, good sleep quality, quitting smoking, limiting alcohol consumption, and being aware of environmental hazards are essential for healthy ageing.

Despite the most direct fall and CV factors involving the physical and clinical domains, issues are also related to psychological and social spheres. Indeed, the fear of falling can lead to loss of confidence, social isolation, and solitude, which are associated with a higher risk of falling [17], developing cardiovascular diseases, and mortality [18].

Based on the main determinants for falls and cardiovascular events, the participants were evaluated through an extended multidimensional evaluation by a specialised team of clinical and social operators. Data on the global health of each individual were collected among several health domains (i.e., mobility, psychology, nutrition, cardiology, and social) to outline a multidimensional health profile of the patient. Each health and social care professional expressed their epicrisis modulated by an interdisciplinary discussion. The screening of the frailty condition included face-to-face interviews for anamnestic information, validated questionnaires, and risk scales (Table 1) to assess the patient’s health status quantitatively, the evaluation of the performance during specific physical tasks, and the execution of clinical exams. Table 2 reports a list of dichotomous variables acquired during the visits. To summarise, the investigated variables consisted of socio-demographic information (e.g., age, gender, living status, and living context), social aspects (quality of relationships, financial aspects, and educational level), anthropometric assessment (weight, height, and body mass index), functional independence in the activities of daily living, mobility and equilibrium investigation, psychological investigation (mood disorders, cognitive status, and psychological attitude), neurological examination (neurological deficit, muscle tone, and hypotrophy), and clinical data (e.g., blood pressure, bio-impedentiometry parameters, ECG exam, cardiovascular risk factors, hearing impairments, visual impairments, and history of previous falls).

#### 2.1.2. Devices and Services Set

The proposed decision algorithm aims to support clinicians in prescribing a health prevention strategy customised to the elders’ needs. The algorithm’s output is represented by the assignment of a personalised set of devices and services, chosen according to the elders’ clinical and social needs, arising from the multidimensional assessment. Table 3 reports the technological devices and the services that make up the database of the reasoning system.

Technological devices are non-invasive and easy-to-use systems that provide continuous assistance to the patient at home and outside. They also monitor health status while improving social inclusion. The main focus is to prevent severe circumstances, such as a fall or acute events (e.g., the onset of cardiac episodes) by enhancing their lifestyle quality and monitoring the related vital signs.

The technological devices were identified based on the type of monitoring (manual or continuous), device (environmental, wearable, portable, etc.), and user compliance during usage. The selected technologies can be adapted to the functional abilities of the elderly, being customisable in the interfaces and settings.

Fall monitoring devices include an ambient sensor, a wearable smartwatch, and a pendant. Due to privacy issues, a radio frequency-based fall ambient detector was preferred. Indeed, optic sensors are perceived to violate privacy due to the feeling of being continuously observed [20]. Older people like unobtrusive radio-frequency devices, despite the problems related to signal coverage range. Thus, multiple sensors should be located in strategic locations of the house when the patient is at higher risk of falls.

Given that older adults falling occurs inside the house almost half of the time, the rooms at higher risk of falling are the kitchen (25%), the bedroom (22%), inner and outer stairs (20%), and the bathroom (13%) [21]. Radiofrequency fall sensors analyse radio frequency signals in the home. They trigger an alert whenever a silhouette immobilised on the ground is detected. They could be ineffective for multiple people living in the room or small to medium-sized pets such as dogs or cats. In the latter case, a false positive alert could be triggered. The proposed fall ambient sensor (by Vayyar^®^) is mounted on the wall or ceiling of the room. It uses a Wi-fi connection for 24/7 monitoring of the elderly. Falls are automatically detected by the system, which sends an SMS to the caregiver for prompt intervention, increasing the safety and well-being of the patient. This solution is suggested for those who spend time at home. Regardless, it is incompatible with the presence of cohabitants or house pets for the reasons mentioned above.

A different solution is provided by MICROTECNO^®^, developing a wearable sensor as a necklace or attached to the belt. It is based on an accelerometer with customisable sensitivity able to automatically detect a fall and trigger an alert via phone call and SMS. It takes advantage of GPS technology to inform relatives and caregivers on the position coordinates of the elderly patient at any time through real-time geolocation. Security zones can also be set up that send a notification whenever the senior enters or exits them. Moreover, the device allows receiving and sending voice calls by pushing a big SOS button. Apple^®^ provides a further solution through a smartwatch able to automatically detect a fall event and alert the emergency services for prompt intervention. When the watch detects an abrupt change in motion and stillness for about 30 s, it alerts the patient with a vibration on the wrist and an audible alarm. It displays an alert message so the person can tap the emergency SOS or reset the alarm if they are okay. This device is not limited to fall detection. Indeed, it has several functionalities for multiparametric monitoring. It is, thus, possible to use an ECG (series 4 and later) to find atrial fibrillation or rhythm alterations, measure heartbeats at rest or during physical activity, and measure the oxygen level in the blood through an infrared sensor (series 6).

Concerning the previous fall detection system, the use of an Apple Watch^®^ requires higher technological abilities from the patient. Moreover, it is not suggested for patients with prostheses in both hands, to not compromise the efficacy of the use of the device itself. It is also not recommended for those who present implanted cardiac devices, such as PMK or ICD, for safety reasons, because dangerous electrical interferences may be generated.

An alternative device to the smartwatch is the pocket-sized KardiaMobile (by AliveCor^®^), which consists of a mobile single- or six-lead ECG device for remote patient monitoring. Everywhere and anytime, this pocket electrocardiograph records ECG and heartbeat. ECG can be shared with the medical doctor to diagnose or manage cardiac anomalies. Even in this case, patients with both hands prostheses, as well as PMK or DFB implanted, are advised against the use of this device. Therefore, since slightly more complex operations are required to be performed with the KardiaMobile to obtain an ECG track, this kind of monitoring is suggested for patients with higher technological abilities.

The blood pressure monitor should also be mentioned to conclude the overview of cardiac monitoring devices. Enrolling the cuff around the upper arm, the Omron^®^ system measures the arterial blood pressure. It detects irregular heartbeats at home or on the go. Results and progress can be saved and followed, which is particularly helpful in managing patients with hypertension.

In contrast, the smart body scale (by Renpho^®^) permits deep body composition analysis by keeping track of body weight, BMI, body fat, water percentages, skeletal muscle, fat-free body weight, muscle mass, bone mass, proteins, etc. Goals can be set, and progress can be tracked so that the patient can be monitored in their weight condition or adherence to a specific diet, for example.

Finally, the tablet device (by MICROTECH^®^) can be used with dual functionality. On one side, as a communication device, it enables the elderly to remain in contact with their relatives and friends and the medical doctor. This device prevents social exclusion and may positively impact the psychological and emotional status of the patient. On the other side, it proposes games and activities for recreational function or cognitive improvement.

It has been mentioned many times that some devices use an app to store and share data or to send notifications and alarms. A smartphone configured as a mobile hotspot can be provided if needed.

These digital devices presented are the core of the study because they help monitor the patient’s status remotely and continuously in terms of physiological parameters, movement patterns, and falls. In some cases, a specific service (Table 3) is offered to the patient to improve their general well-being.

Social support services include home care if the patient encounters difficulties because of architectural barriers:

- A social operator for animation and entertainment activities.
- It may be proposed to an elderly person who is particularly active with no mobility limitations that they volunteer in their community. It is an excellent opportunity to share their experiences, help needy people, and make new friends.
- Some kind of physical activity at low or moderate intensity is essential in old age and is associated with many health benefits.

Psychological support may be indicated for patients with fragile or critical psychological status. Group therapy for listening and talking sessions or occupational therapy (e.g., gardening, yoga, breathing, and low-impact exercise) engage the elderly in group activities with other peers. It favours social interaction, participation, and positive emotional and behavioural responses, improving the quality of life.

A transport service is suggested for people with walking mobility problems or who are not car owners and would like to reach desired areas of interest.

Finally, besides social services, there are also healthcare services related to follow-up visits for the investigation of specific conditions: neurological, nutritional (divided into weight control, sarcopenia, and diet consultation), renal functionality, hearing, vision, and fall-related examinations.

### 2.2. Data Preprocessing

The decisional algorithm takes as inputs the previously described variables from the multidimensional assessment (i.e., the social and clinical characteristics of the elderly) and the selected product and services. As output, the algorithm produces a specific and personalised kit based on user needs.

Data preprocessing is a crucial step for subsequent analysis. First, all data from the multidimensional assessment were classified as continuous and dichotomous variables. Continuous variables (e.g., TUG, MBI, GDS, and MNA) were acquired as numerical values, whilst dichotomous variables (e.g., previous falls, previous syncopes, and recent loss) were coded in binary form as 0 or 1 based on the absence or the presence of the deficit, respectively. Table 1 and Table 2 report all the variables included in developing the decisional-support tool for personalised product-service assignment. Specific cutoffs were adopted for driving the tailored assignment process (Table 1 shows the cutoffs for the continuous variables).

### 2.3. Decisional Analysis and Algorithm Development

The algorithm was conceived as a decision tree. This decision-support tool, which uses a tree-like model, contains conditional control statements. Using sequences of binary divisions (dichotomy approach) drives the assignation of products and services (available for the study) to each user, depending on their clinical and social requirements.

First, information, education, and communication are recommended to all. This premise of the algorithm consists in providing advice and interventions to increase trust and awareness about everyday risks indiscriminately to the whole target elderly population. Patients can be informed about the environmental and health risks of falls and the behaviours that should be adopted to prevent them. Moreover, this advice concerns cardiovascular prevention, nutritional and lifestyle habits, and, thus, periodic screening.

Additionally, the algorithm contains different investigation areas that manage and integrate patient information, elaborate it, and produce a proper output for each patient. Conditions are tested, and other results are proposed to the patient depending on the response. As extensively described in Section 2.2, these outputs can include:

- Digital devices, with different functionalities and technological impacts depending on the particular ability of the patient;
- Social and healthcare services offered and suggested to the elderly to improve their well-being and quality of life in the specific needed area.

In addition to input and output variables, boundary conditions are set in the decisional analysis to make the process more efficient. The patients are classified into two categories based on their predisposition and inclination to the technology. Suppose they are part of the “active” group, named class (A). In that case, they may be associated with high technological impact devices. In contrast, if they belong to the “passive” group, named class (B), they may be assigned to the low-technology, easy-to-use devices. Additionally, specific function features of the devices (e.g., interferences, particular shortcomings, etc.) are taken into account in the decision-support logic.

**Figure 2:**
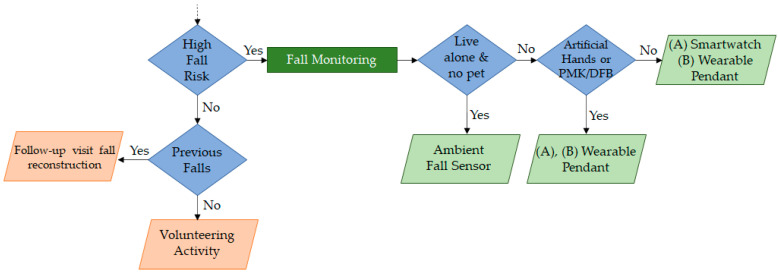
Decisional flowchart—excerpt of fall domain.

A flowchart, a simple and intuitive graphical representation, has been employed to explain the decisional process at the base of the algorithm. The entire algorithm flowchart is available as supplementary material in Figure S1. Figure 2 shows an excerpt of the decision analysis related to the fall domain.

In this excerpt, the algorithm evaluates the fall risk. If the fall risk is low (based on the combination of the previously illustrated thresholds TUG, BBS, WHS, and MBI), the presence of eventual previous falls is investigated. If the patient has fallen, a follow-up visit is recommended. Otherwise, it is suggested that they perform volunteering activities to keep them healthy and active. A fall monitoring system is indicated if the fall risk is high. Other patient characteristics are investigated to define the best device fitting their need. The ambient fall sensor is indicated if the patient lives alone and does not have pets at home. Instead, if the patient lives with someone and the Vayyar^®^ cannot be provided, then the presence of PMK/DFB and prostheses in the hands is assessed. If they are absent, then the smartwatch or the wearable pendant is suggested based on the patient’s aptitude for technology (class (A) or (B)). Otherwise, the wearable pendant is provided in both cases (patients cannot wear the smartwatch with artificial hands or PMK/DFB). Then, the cardiological investigation area receives input from the fall risk evaluation data. In this way, if the patient has already been recommended a smartwatch for fall monitoring, it is confirmed whether cardiological monitoring is required (avoiding the assignation of another device).

### 2.4. Algorithm Implementation

The algorithm was implemented with Visual Basic for Applications, an event-driven programming language to record, create, and edit subroutines. VBA uses functions and objects to perform an automated set of operations. VBA was employed to exploit the advantages of simplicity and immediacy of being integrated into Microsoft Office Excel. Indeed, the MOSAICO software platform provides the patients’ database in Excel. Through VBA, the decisional workflow was converted into code interacting with the patients’ database through specific iterated logic. The decision-support logic was composed to be cautiously conservative in case of a lack of information. In this manner, the algorithm avoids the eventual dangerous lack of devices or services assignment. The programme keeps the patients’ data from an Excel sheet. It presents, as output, the assignment for each patient of the needed devices and services. A macro allows automatically running the code for each patient.

### 2.5. Validation

The algorithm’s reliability concerning the user device/service matching was tested by statistical analysis in terms of sensitivity and specificity. These mathematical parameters describe, in general, the accuracy of a test in reporting the presence or absence of a determined condition. Additionally, they are standard methods to assess a diagnostic test’s accuracy, validity, and ability to accurately measure what it is meant to [22,23].

The test allows discriminating between four categories: (i) true positive (TP), (ii) false positive (FP), (iii) true negative (TN), and (iv) false negative (FN).

The sensitivity (S) (true positive rate) and specificity (Sp) (true negative rate) can be calculated as respectively reported in Equations (1) and (2):

(1) $$\mathrm{Sensitivity}=\frac{\mathrm{TP}}{\mathrm{TP}+\mathrm{FN}}$$

(2) $$\mathrm{Specificity}=\frac{\mathrm{TN}}{\mathrm{TN}+\mathrm{FP}}$$

In this work, the test is represented by the proposed algorithm. The condition assessed was the patient’s candidature (or not) for a fall or cardiac monitoring device and the neurological medical examination. This condition was tested against the clinical assessment to evaluate and validate the algorithm’s performance. The sensitivity indicates the probability of the algorithm correctly assigning a patient as eligible for a fall/cardiac monitoring device or the neurological visit if they require them, based on the clinician’s opinion. The specificity represents the probability of the algorithm assigning a candidate for a fall/cardiac device or neurological visit when the patient does not need any monitoring or a medical examination. False positives and negatives are computed to evaluate the algorithm misassignments (i.e., FP: a cardiac/fall device or neurological visit is assigned when not needed; FN: a cardiac/fall device or neurological visit is not set when required).

## 3. Results and Discussion

### 3.1. Study Participants

To validate the automatic decision-support algorithm, a sub-sample of fifty elderly patients from the study by [24] were enrolled. All subjects signed the informed consent form and agreed to participate in the survey after a careful and extensive explanation of the scope, procedures, potential benefits, and risks. They were selected based on the inclusion and exclusion criteria shown in Table 4.

The target population, coming from twelve municipalities of the seismic crater areas of Central Italy, meets two inclusion criteria: having an age over 75 and being a grade I, II, or III according to the Rankin modified scale, which means a good-to-moderate level of autonomy. Additionally, it should be mentioned that comorbidities and pathologies to organs and systems (e.g., cardiological, vascular, endocrine-metabolic, neurological, sense organs, renal, hepatic, gastrointestinal, respiratory, bones, and joints problems) and joint prostheses or implanted cardiac devices are not exclusion criteria. The only exclusion criteria consist of the survival diagnosis of less than one year and severe cognitive impairment that would not guarantee adequate and reliable participation in the experimentation.

The sample resulted in 16 males and 34 females, with a mean age of about 83. The data employed for the algorithm validation were collected during various sessions of multidimensional assessment by a multidisciplinary team of social and healthcare specialists within the MOSAICO project.

### 3.2. Statistical Description of Participants

Table 5 and Table 6 report the statistical description of the target population. Table 5 shows an overview of the patients’ characteristics. In particular, 80% of participants were not familiar with the use of technology and were assigned to the passive class. Only 22% had a smartphone, and 36% had a Wi-fi connection at home. Even though 76% had social relationships, half lived alone (28% had a recent familiar loss). Most of them suffered from hypertension (98%), 80% were cardiopatic, and 24% had a previous syncope. Additionally, half of them had experienced at least one previous fall.

Table 6 contains mean values and standard deviations of questionnaires and tests for physical performance (MBI, TUG, BBS, and WHS), psychological evaluation (GDS, Zung anxiety scale, and corrected MMSE), and nutritional assessment (handgrip, muscle mass, and MNA).

Concerning the physical conditions, mean values show high performance in walking 3 m (TUG), low risk of falling (BBS), minimal dependence (MBI), and most-limited community (WHS). Regarding the psychological domain, participants had mild–moderate depression (GDS) and mild anxiety levels (Zung). Indeed, while anxiety prevails in the younger population, depression is predominant in the elderly. The mean values of the cognitive functional test were barely low (low performance for the corrected MMSE ≤ 24). The muscle mass was over the cutoff values both in males and females. The handgrip strength was over the cutoffs only for females, indicating that the overall muscle strength in men is reduced. Concerning the nutritional assessment, participants had a normal nutritional status (MNA).

### 3.3. Algorithm Validation

The algorithm validation results are presented in Table 7.

According to the algorithm definition, 68% of the target population (*n* = 50) requires fall monitoring, compared to the 50% proposed by the clinical evaluation. Matching assignation, which results when the algorithm’s output coincides with the clinician’s output, is reported in 78% of the cases. Non-matching assignations refer to two situations: the patients addressed to a fall monitoring device by the algorithm but not by the clinician (FP) and those who were not assigned candidates by the algorithm but assigned candidates by the clinician (FN).

This second case represents a more complex situation since the patients who need to be followed by fall monitoring are instead ignored. However, from this study, only 2% of cases fall into this group of patients. This meagre percentage corresponds to just one patient.

Moreover, sensitivity and specificity have been computed to understand the developed algorithm’s potential and reliability. Specifically, for fall monitoring assessment, the algorithm shows high sensitivity (96%), which means it does not miss needy patients. Medium specificity (60%) means that the algorithm is not highly performant in ruling out people who are not needy. Therefore, this second aspect should be improved.

Regarding the cardiac assessment, 84% of the target population requires cardiac monitoring per the algorithm method. In comparison, the clinician proposed a 56% percentage. This corresponds to a matching assignation in 64% of the cases. Again, considering the rate of FN that contributes to the non-matching assignation, the number of people who would require cardiac monitoring but instead are ignored by the algorithm can be evaluated. The value is 4%, slightly higher for the fall monitoring assessment. Regardless, a meagre percentage indicates that the algorithm incorrectly classified only two patients.

In this case, the sensitivity is again high (93%), which means the algorithm correctly identifies patients needing cardiac monitoring. However, the specificity is very low (27%) because many patients were addressed to cardiac tracking even if they did not need it.

To take stock of these results, high sensitivity and low specificity values support the conclusion that the present algorithm adopts a more preventive and protective approach. It led to a higher percentages of patients assigned to fall or cardiac monitoring than that identified by the clinical evaluation. This conservative strategy, however, does not exclude and correctly permanently recognises needy patients, apart from very low percentages.

Moreover, Table 7 compares the assignation of the neurological visit between the algorithm and clinical evaluations for the algorithm validation. The algorithm, which assigns the neurological visit in the presence of severe dementia (MMSE) or history of precedent ictus, assigns 22% of the patients as candidates, compared with the 26% proposed by the clinician. This corresponds to a matching assignment of 88%. In this case, the number of needy patients ignored by the algorithm increases to four persons (8%). This result can also be appreciated by the low sensitivity (69%) and the high specificity (95%) estimated in the statistical analysis.

**Figure 3:**
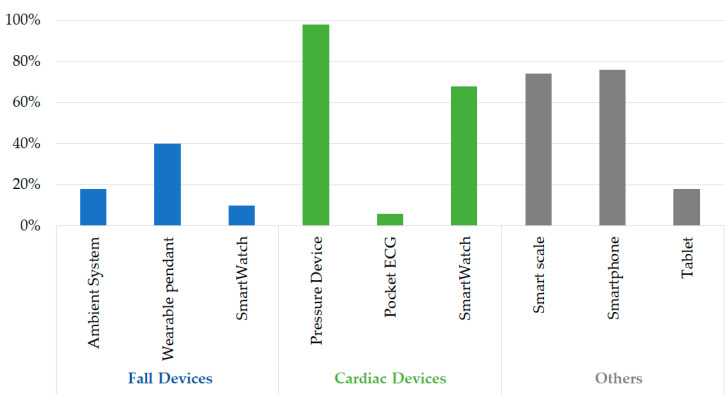
Assignation percentages of different monitoring devices.

The assignation percentages referring to each specific device are reported in Figure 3.

The wearable pendant (low-technological impact device) was given to 40% of the 68% of falls monitored. The smartwatch for cardiac monitoring was given to 68% of the cases out of the 84% of cardiac candidates observed. This finding is understandable since 80% of the patients belong to the passive class, which means a low predisposition to technology. In comparison, just 20% fall into the active category, indicating a greater inclination toward technology.

Finally, significant percentages emerge from the smart scale (74%) and the smartphone assignations (76%). The latter means that patients would require an internet connection to use the assigned devices, but they do not have it (lack of Wi-fi and need for a smartphone as a hotspot).

**Figure 4:**
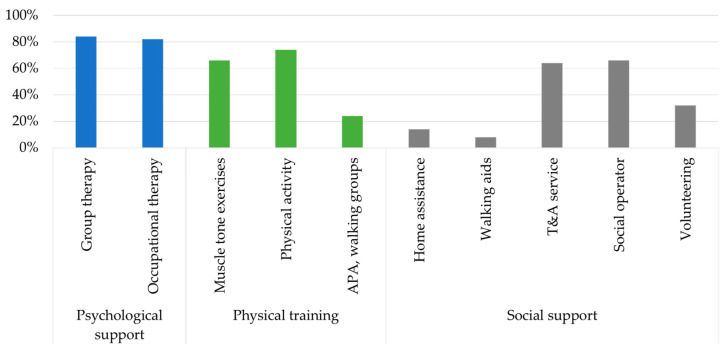
Assignation percentages of services.

**Figure 5:**
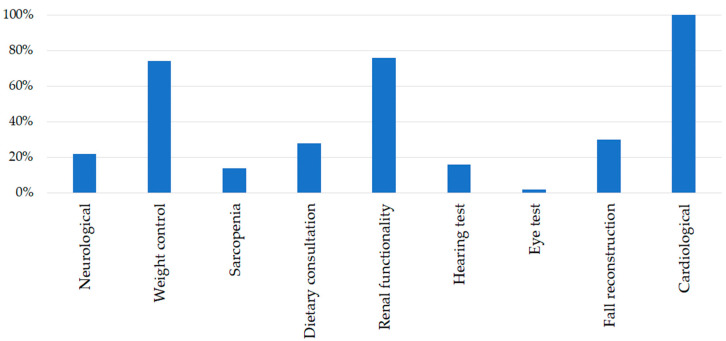
Assignation percentages of follow-up visits.

Figure 4 and Figure 5 show results concerning the services and follow-up visits considered in the algorithm and assigned to the patients.

Significant percentage values (Figure 4) were recorded for psychological support in both group therapy (84%) and occupational therapy (82%). This highlights the need for an intervention in response to a widespread critical depressive status among the elderly. Therefore, many of them were prompted to improve their physical activity (74%) by performing regular training. In particular, those with risk or confirmed sarcopenia were indicated for muscle tone exercises (66%) that can increase muscle mass and reduce muscle weakness. High scores were also obtained in transportation and accompaniment (64%) services and social worker (66%) service, conceived as a person who keeps the elderly company and offers entertainment activities.

The most needed visits (Figure 5) were cardiological (100%), renal functionality examination (76%), and weight control (74%). The cardiological visit results were assigned to the whole target population. Even if patients do not report any precedent cardiac event or chronic renal insufficiency (CRI), almost everybody presented at least one cardiovascular risk factor (i.e., smoking, diabetes, familiarity, hypercholesterolemia, or obesity). Not negligible is the fall reconstruction visit (30%), assigned to those classified as low fall risk according to the quantitative scales but who have experienced single or frequent falls in the recent past that merit consideration.

## 4. Conclusions

This paper proposes a decision-support automatic algorithm to help clinicians find the best products and services tailored to elders’ social and clinical needs. Its multidimensional nature investigates multiple aspects of the elders’ health, not limited to the functional and mobility domain, for more accurate identification of their deficits. The product/service user matching aims to encourage elders to use digital devices to monitor their health status in a preventive way while taking advantage of social inclusion activities.

Promoting proactive behaviour focused on preventing acute events rather than their treatment provides multiple benefits. Indeed, it can encourage awareness of the elders’ health status to preserve independent living or to improve their well-being. The National Health System could spare financial resources to promote reduced hospitalisation and better service quality. Finally, it is a step towards the digitalisation of society since using such technological devices involves elders, caregivers, and general practitioners.

The algorithm presented in the paper was conceived as a decision tree. It drives the assignation of products and services to each user, depending on their clinical and social requirements. The products refer to digital devices with different functionalities and technological impacts depending on the particular ability of the patient. Social and healthcare services aim to improve elders’ well-being and quality of life in the specific needed area.

The algorithm’s reliability concerning the user device/service matching was statistically evaluated over a sample of 50 users in terms of sensitivity and specificity. Its output was compared to clinicians’ decisions during the multidimensional evaluation to validate the algorithm. The algorithm reported a high sensitivity (96% for fall monitoring and 93% for cardiac tracking) and a lower specificity (60% for fall monitoring and 27% for cardiac monitoring).

This result highlights the preventive and protective behaviour of the algorithm. However, it misclassified a high percentage of patients as needy, contrasting with the clinicians’ assessment. Thus, to improve the algorithm’s reliability, a larger sample size should be considered. Moreover, 1-year monitoring of the study participants could validate the algorithm’s development procedure by assessing its efficacy in terms of non-worsening health status, early diagnosis of disease, and improved life quality. Future research could be devoted to developing an automatic software tool with enhanced accessibility, availability (based on a mobile app for Android/IOS smartphones), and usability (ease of use by general practitioners).

## Figures and Tables

**Table 1:** Continuous variables and associated thresholds assumed for the development of the algorithm.

Test	Description	Score	Cutoff Points
Timed up and go (TUG)	It is performed to assess the fall risk of a user by measuring the amount of time a person is required to stand up from a chair, walk 3 m, turn around, walk back to the chair, and sit down.	N/A	≥20 s high falls risk
Berg Balance Scale (BBS)	It is a 14-item scale that evaluates the balance ability of a person performing a series of tasks. Each item consists of a five-point ordinal scale ranging from 0 to 4, with 0 indicating the lowest and 4 the highest level of function.	It ranges from 0 to 56 where: Low fall risk: 41–56 Medium fall risk: 21–40 High fall risk: 0–20	≥41 low falls risk
Walking Handicap Scale (WHS)	It determines the mobility quality in the home and the community contexts across 6 categories.	Physiological walk: 1 Domestic walk with limitations: 2 Domestic walk without limitations: 3 Social walk with major limitations: 4 Social walk with some limitations: 4 Social walk without limitations: 6	<5 limited walk
Modified Barthel Index (MBI)	A validated scale is used to measure the performance in activities of daily living (ADL) along 10 items.	It ranges from 0 to 100 where: Total dependence: 0–24 Severe dependence: 25–49 Moderate dependence: 50–74 Mild dependence: 75–90 Minimum dependence: 91–900 Independence: 100	<91 dependence
Geriatric Depression Scale (GDS)	It consists of a 30 question-survey designed to rate depression in elderly patients. It is not recommended to use GDS when the patient is characterised by severe dementia.	It ranges from 0 to 30 where: No depression: 0–10 Low-moderate: 11–16 Severe: ≥17	>10 depression
Mini-Mental State Examination (MMSE)	A validated tool to assess cognitive function through seven domains (orientation to time, orientation to place, three-word registration, attention and calculation, three-word recall, language, and visual construction).	It ranges from 0 (severe cognitive impairment) to 30 (no cognitive impairment). An MMSE score greater or equal to 24 is considered normal cognitive function, while scores less than 24 indicate cognitive impairment. Then, the MMSE score can be corrected according to age and education.	≤15 severe dementia
Zung Anxiety	Zung anxiety scale presents 20 items that ask how the patient has felt or behaved during the past several days. The items are judged on a four-points system, considering severity. Four anxiety categories are identified; the higher the total score, the higher the anxiety level.	It ranges from 0 to 80 where: Low anxiety level: 20–31 Low-medium anxiety level: 32–43 Medium anxiety level: 44–55 Medium-high anxiety level: 56–67 High anxiety level: 68–80	>40 anxiety
Mini Nutritional Assessment (MNA)-Short form	It allows a rapid assessment of the nutritional status of the elderly. The shortened version consists of six items incorporating anthropometric measurements, dietary intake, and global- and self-assessment components.	The total score ranges from 0 to 14 where: Malnourished: 0–7 At the risk of malnutrition: 8–11 Normal nutritional status: 12–14	<12 abnormal nutritional status
Body Mass Index (BMI)	It refers to the nutritional status of adults. It is defined as a person’s weight in kilogrammes divided by the square of the person’s height in metres (kg/m^2^).	Underweight: less than 18.5 Normal weight: 18.5 to 24.9 Overweight: 25 to 29.9 Obesity class I: 30 to 34.9 Obesity class II: 35 to 39.9 Obesity class III: above 40	<18.5 or >24.9 abnormal nutritional status

**Table 2:** Dichotomous variables acquired during the multidimensional health assessment.

Dichotomous Variable	Description
Previous falls	History of previous falls defined as “unexpected events in which the user come to rest inadvertently on the ground, floor, or lower level”
Walking aids	Use of any device designed to assist walking or otherwise improve the mobility of people (e.g., canes, crutches, or walkers)
Living alone	A person living alone in their household, independent from marital status, the number of children, friends, and relatives
Social relationship	Sporadic relationships with family, friends, and neighbours
Conflicting relations	Quarrels and conflicts that broke the relationship
Recent loss	Death of a loved person in the past seven months
Handgrip strength	An indicator of overall muscle strength. It is measured as the amount of static force in kilogrammes that the hand can squeeze around a dynamometer. The mean value of three tests was acquired, and the highest value between the two hands was considered. Cutoff values are <27 kg (male) and <16 kg (female) [19]
Muscle mass	The amount of skeletal muscle mass assessed through the BIA. Cutoff values are <20 kg (male), and <15 kg (female) [19]
Normal hydration	Assessment of total body water through bioimpedentiometry
Physical inactivity	Sedentary lifestyle
Cardiovascular risk factors	Presence of at least one of the following risk factors: smoke, diabetes, hypercholesterolemia, obesity, familiarity
Hypertension	Occurs when the systolic blood pressure measurements on two days are ≥140 mmHg and the diastolic blood pressure readings on both days are ≥90 mmHg
Previous syncope	Defined as a transient loss of consciousness and inability to maintain the postural tone, followed by spontaneous recovery. It is acquired based on anamnestic data of the user
Previous cardiovascular event	Being subjected to a previous cardiovascular event such as cardiopathy, atrial fibrillation, acute myocardial infarction, chronic ischemic cardiomyopathy, stroke, or heart failure
Kidney failure	Creatinine clearance level 30–59 mL/min
PMK	Having a pacemaker device
ICD	Having an implantable defibrillator
House pet	Pet ownership
Wi-fi	Presence of an Internet connection within the house
Several floors home	Having a house with multiple floors
Elevator	Presence of an elevator or a stairlift
Car owner	Owning a car and being able to drive it
Eye deficit	Refers to the presence of sight deficit assessed based on anamnestic data of the user
Vision eyewear	Eyeglasses for vision correction
Hearing deficit	It refers to the presence of a hearing deficit assessed during the interview with the person
Hearing aids	Wearing assistive listening devices
Smartphone	Having a personal smartphone for communication
Hand prosthesis	Having at least a hand prosthesis

**Table 3:** Products (left) and services (right) output of the decision-support algorithm.

Type	Products		Type	Services
Wearables	ECG (Apple^®^), Pulse oximetry (Apple^®^), Fall detection (Microtecno^®^, Apple^®^)		Social support	Home care, animation, walking aids, volunteering, physical activity
Non-wearables	ECG (AliveCor^®^), Blood pressure (Omron^®^), Body composition smart scale (Renpho^®^)		Psychological support	Group therapy, occupational therapy
Ambient	Fall detection (Vayyar^®^)		Transport	Mobility of the elders
Communication	Tablet (MICROTECH^®^) and smartphone (Apple^®^)		Follow-up visits	Neurological, nutritional, renal functionality, hearing, vision, fall reconstruction

**Table 4:** Inclusion and exclusion criteria for the participant sample recruitment.

Type	Criteria
Inclusion	Over 75 years old
	Grade I, II, III of Rankin modified scale
Exclusion	Severe cognitive impairments
	Severe or terminal illness, with a survival diagnosis of fewer than 12 months

**Table 5:** The statistical description of the target population—Dichotomous variables.

Item	Yes		Item	Yes
Technological Class:			Living alone	50%
*Active*	20%		Recent loss	28%
*Passive*	80%		Social relationships	76%
House pet	30%		Normal hydration	24%
Wi-fi	36%		Smoking	26%
Several floors home	60%		Hypertension	98%
Car owner	42%		Diabetes	16%
Prostheses:			Familiarity	34%
*Hand*	2%		Hypercholesterolemia	62%
*Knee*	6%		Smoking	26%
*Hip*	4%		Obesity	38%
*Shoulder*	4%		Cardiopathy	80%
Eye deficit	54%		Atrial Fibrillation	20%
Hearing deficit	48%		Acute Myocardial Infection	10%
Smartphone	22%		Chronic Ischemic Cardiomyopathy	16%
Education level:			Ictus	6%
*Elementary school*	48%		Heart failure	2%
*Intermediate school*	10%		PMK	4%
*High school*	6%		DFB	4%
*University*	4%		Previous syncope	24%
Previous falls	50%		Physical inactivity	24%
Walking aids	36%		Chronic Renal Insufficiency	18%

**Table 6:** The statistical description of the target population—Continuous variables.

Questionnaire/Test	Mean ± Dev. Std	Questionnaire/Test	Mean ± Dev. Std
MBI	94.46 ± 15.13	Handgrip	17.14 ± 7.81 [kg]
TUG	17.00 ± 9.31 [s]	Male	17.74 ± 7.73 [kg]
BBS	44.94 ± 12.38	Female	17.16 ± 7.45 [kg]
WHS	4.66 ± 1.34	Muscle mass (BIA)	22.43 ± 6.34 [kg]
GDS	12.06 ± 7.65	Male	23.05 ± 6.32 [kg]
Zung anxiety	34.46 ± 6.53	Female	22.03 ± 6.23 [kg]
Corrected MMSE	23.22 ± 5.11	MNA	12.41 ± 1.91

**Table 7:** Validation results—Comparison of fall, cardiac monitoring, and neurological visit assignment between algorithm and clinician evaluations.

	Algorithm	Clinician	Matching	FN	Sensitivity	Specificity
Fall monitoring	68%	50%	78%	2%	96%	60%
Cardiac monitoring	84%	56%	64%	4%	93%	27%
Neurological visit	22%	26%	88%	8%	69%	95%

## Data Availability

The data presented in this study are available within the paper and supplementary material (Figure S1).

## Supplementary Materials

The following supporting information can be downloaded at: https://www.mdpi.com/article/10.3390/s23041836/s1, Figure S1: Decision-support algorithm for automatically assigning devices and services tailored to seniors’ clinical and social needs.
