# Supplementary material for: Healthy Ageing: A Decision-Support Algorithm for the Patient-Specific Assignment of ICT Devices and Services
Source: Sensors (Basel). 2023 Feb 7;23(4):1836. doi: 10.3390/s23041836 (PMC9963385; doi:10.3390/s23041836)
Supplement: Supplementary file 1 [file sensors-23-01836-s001.zip › sensors-2145731-supplementary.pdf]

Supplementary Materials

Figure S1 Decision-support algorithm for automatically assigning devices and services tailored to seniors' clinical and social needs. The algorithm's complexity requires it to be decomposed into six parts (i.e., images) to be represented. Still, it must be read as a single one. The 1-to-7 numbers in the small grey circles indicate the connections between subsequent parts of the same algorithm. They are just a trick to report the algorithm in its entirety and have no logical meaning. Legend: Orange ovals: start and end of the algorithm; pale blue rhombuses: decisional blocks; yellow parallelograms: output services (clinical or social); green parallelograms: output devices; orange rectangles: fall and cardiac monitoring. Abbreviations: Body Mass Index (BMI), Mini Nutritional Assessment (MNA), Adapted Physical Activity (APA), Pacemaker (PMK), Defibrillator (DFB), Chronic Renal Insufficiency (CRI).

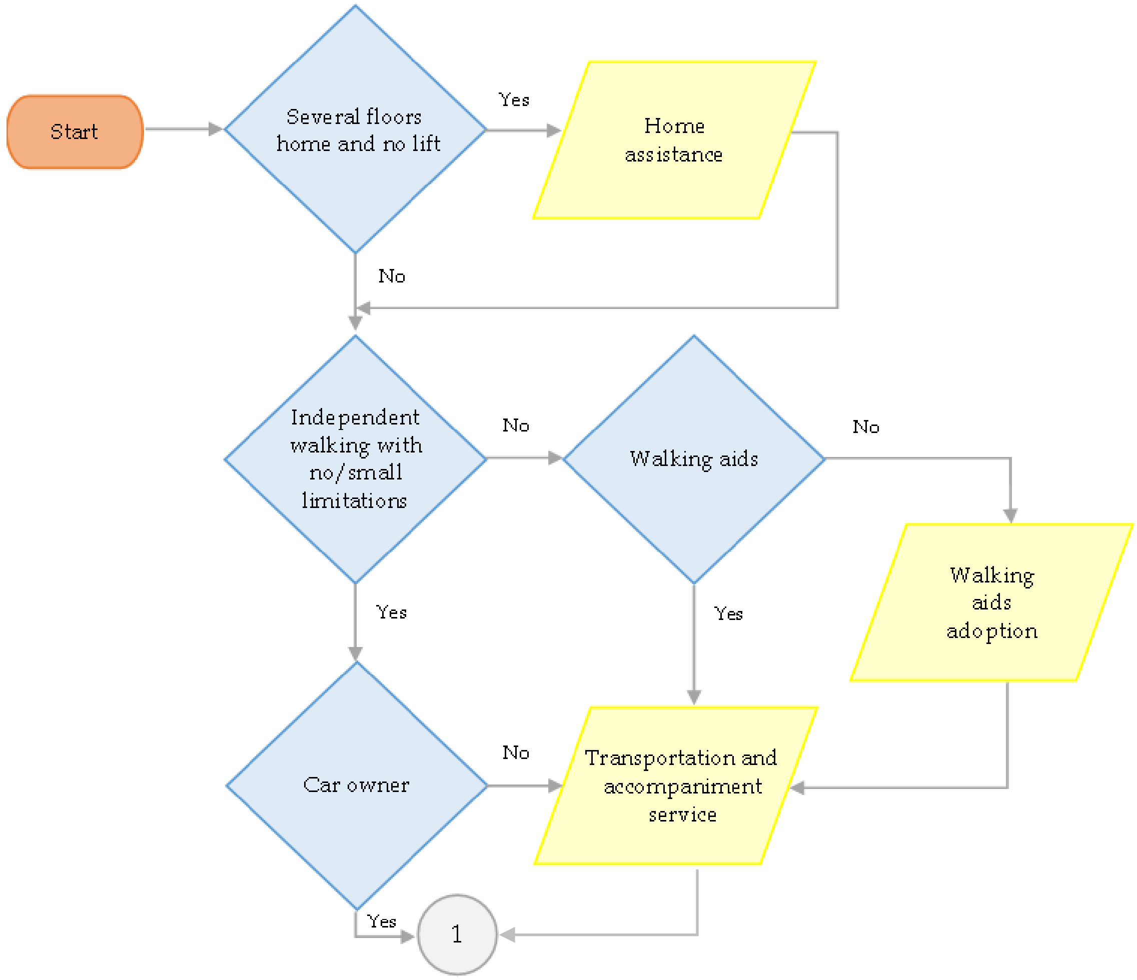

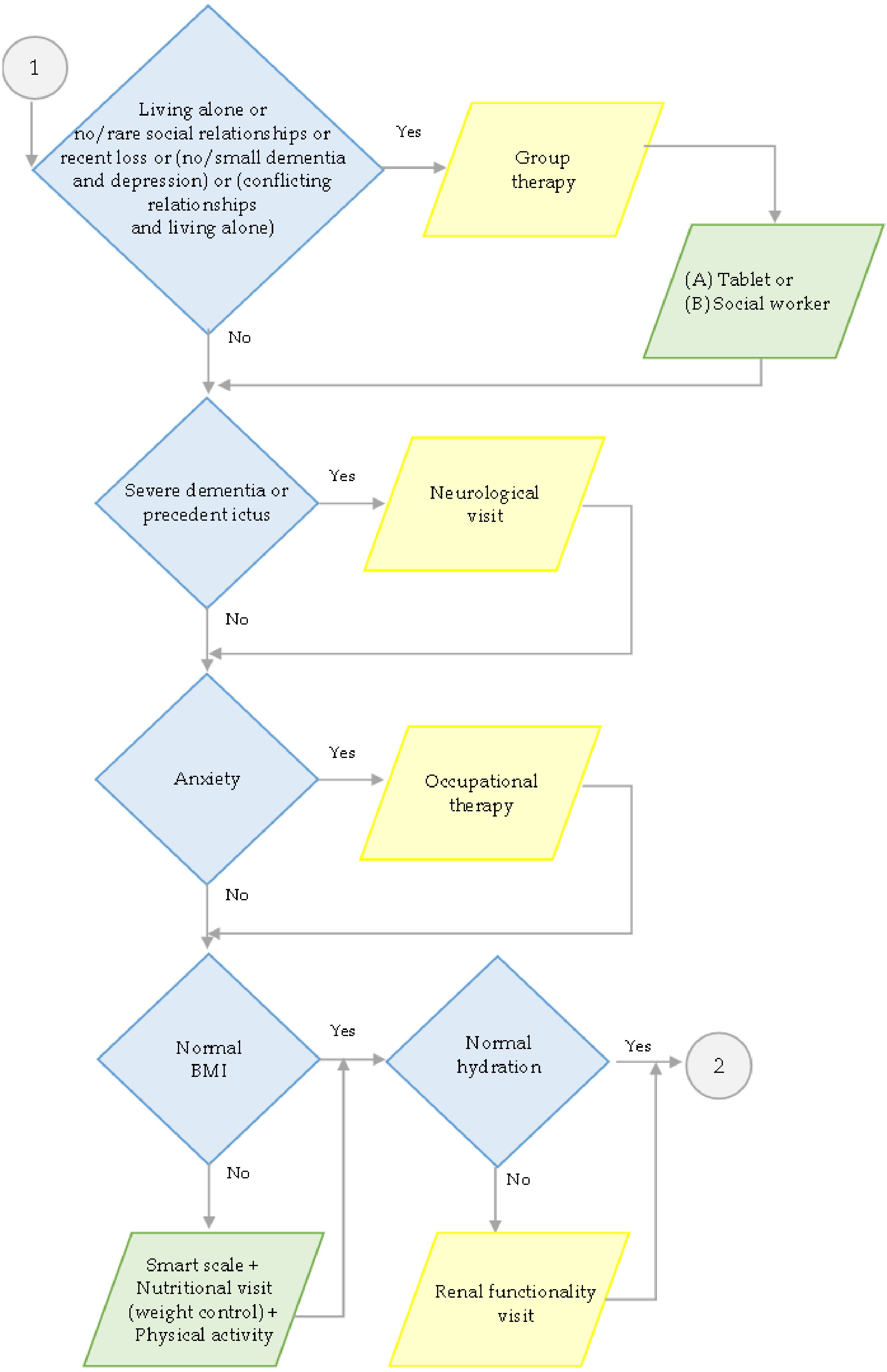
1
Living alone or no/rare social relationships or recent loss or (no/small dementia and depression) or (conflicting relationships and living alone)
Yes
Group therapy
(A) Tablet or
(B) Social worker
No
Severe dementia or precedent ictus
Yes
Neurological visit
No
Anxiety
Yes
Occupational therapy
No
Normal BMI
Yes
Normal hydration
Yes
2
No
Smart scale + Nutritional visit (weight control) + Physical activity
No
Renal functionality visit

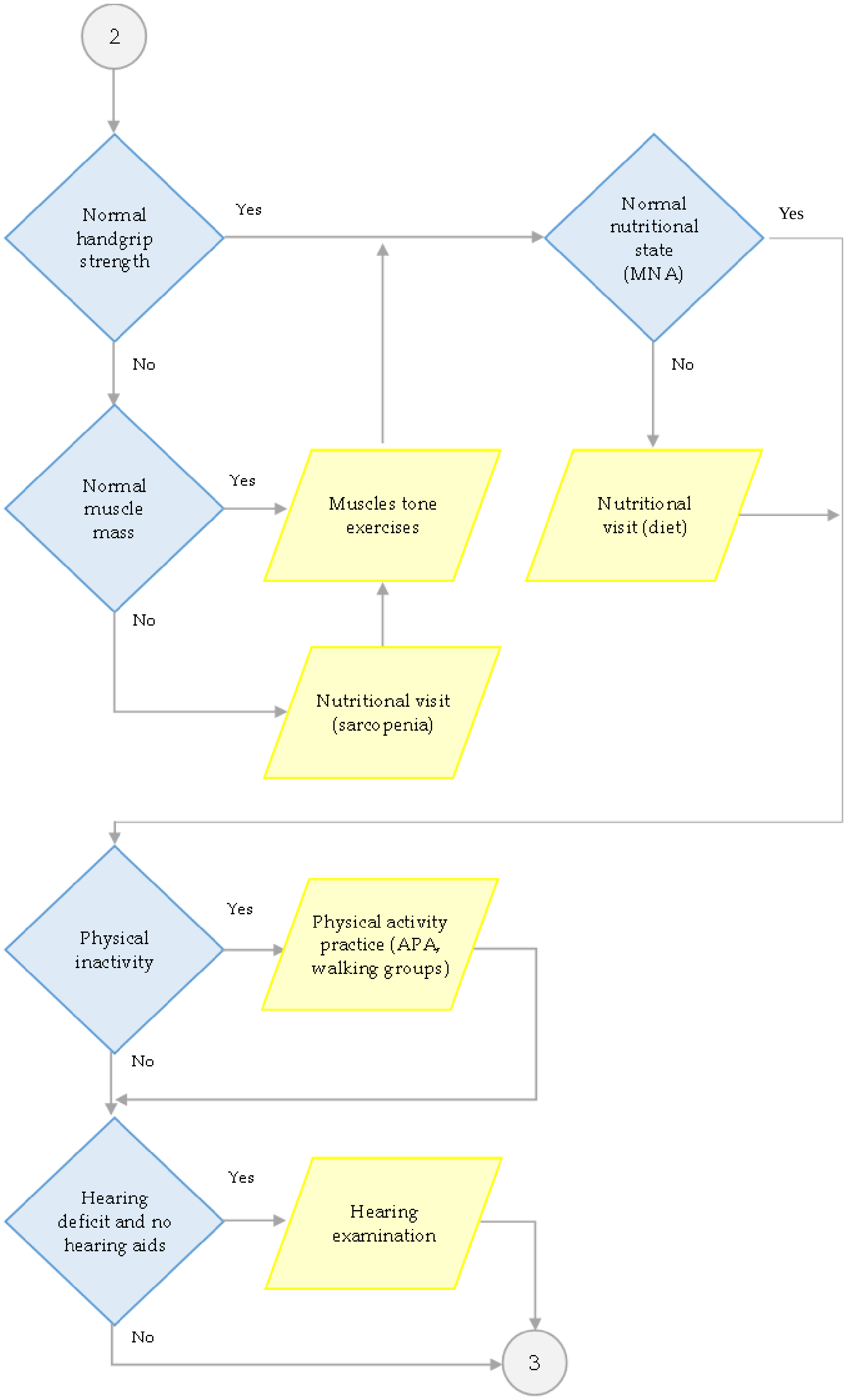
2
Normal handgrip strength
Yes
No
Normal nutritional state (MNA)
Yes
No
Normal muscle mass
Yes
No
Muscles tone exercises
Nutritional visit (diet)
Nutritional visit (sarcopenia)
Physical inactivity
Yes
No
Physical activity practice (APA, walking groups)
Hearing deficit and no hearing aids
Yes
No
Hearing examination
3

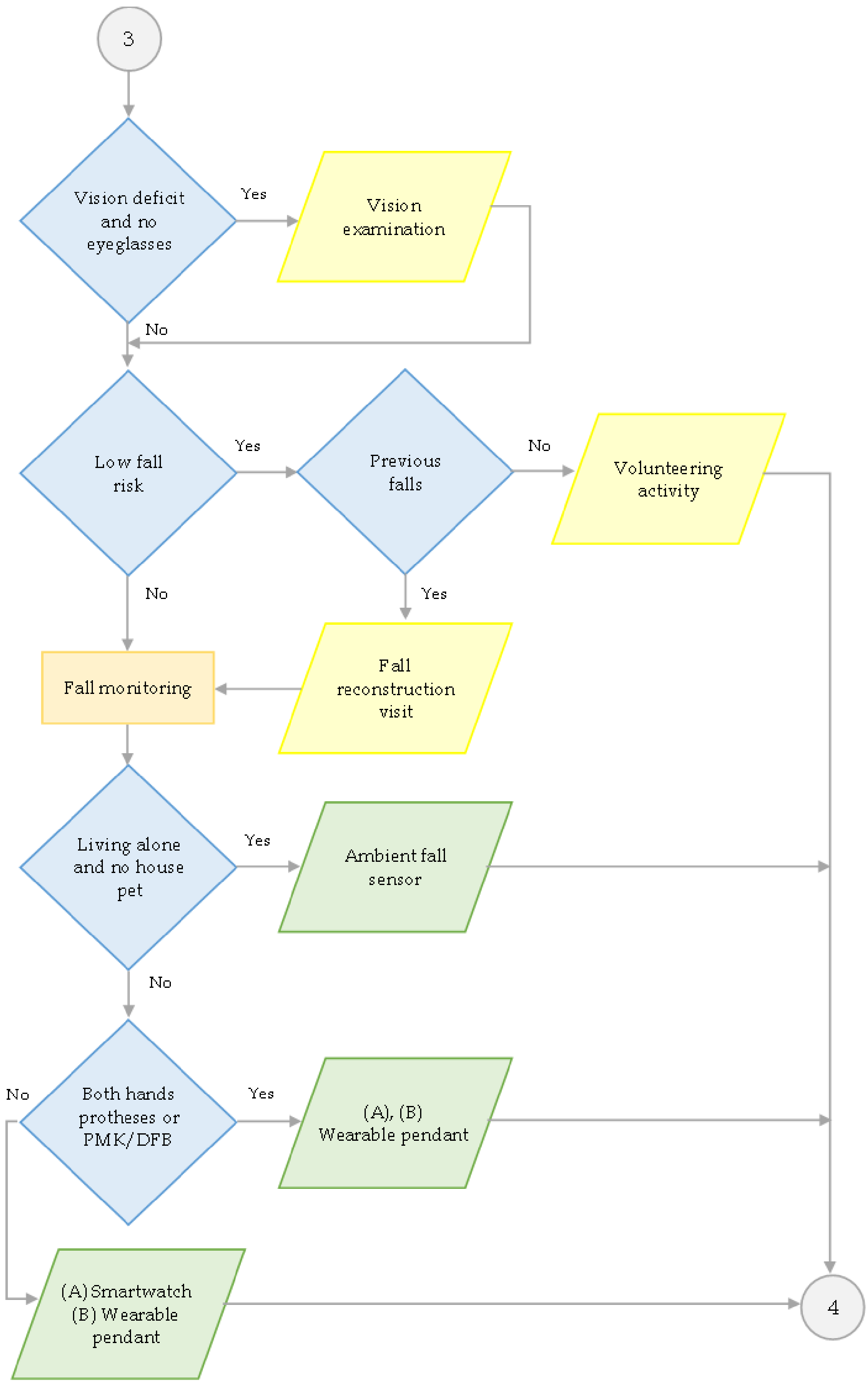
3
Vision deficit and no eyeglasses
Yes
Vision examination
No
Low fall risk
Yes
Previous falls
No
Volunteering activity
No
Yes
Fall monitoring
Fall reconstruction visit
Living alone and no house pet
Yes
Ambient fall sensor
No
No
Both hands protheses or PMK/DFB
Yes
(A), (B) Wearable pendant
(A) Smartwatch
(B) Wearable pendant
4

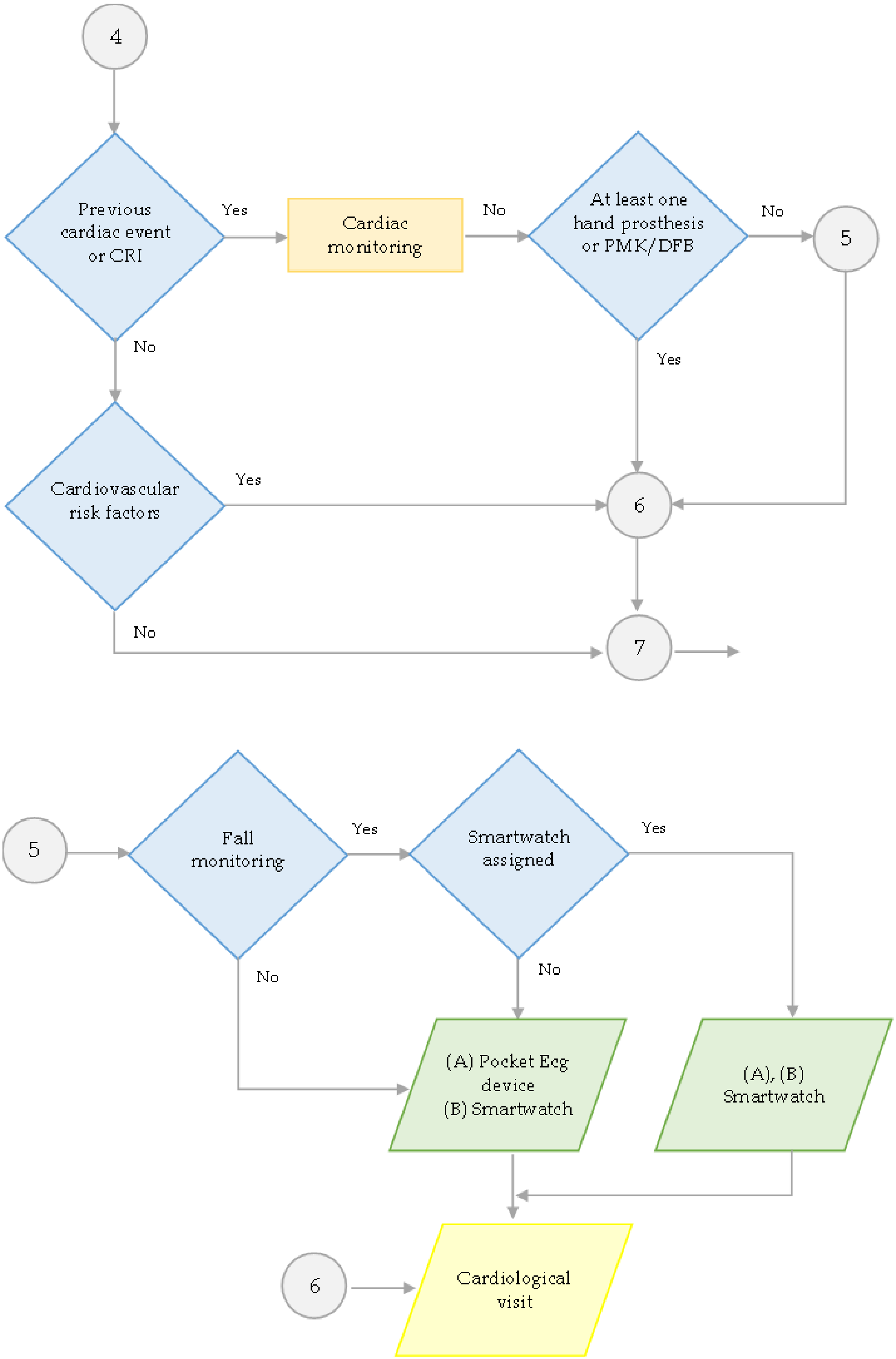
4
Previous cardiac event or CRI
Yes
Cardiac monitoring
No
At least one hand prosthesis or PMK/DFB
No
5
No
Yes
Cardiovascular risk factors
Yes
6
No
7
5
Fall monitoring
Yes
Smartwatch assigned
Yes
No
No
(A) Pocket Ecg device
(B) Smartwatch
(A), (B) Smartwatch
6
Cardiological visit

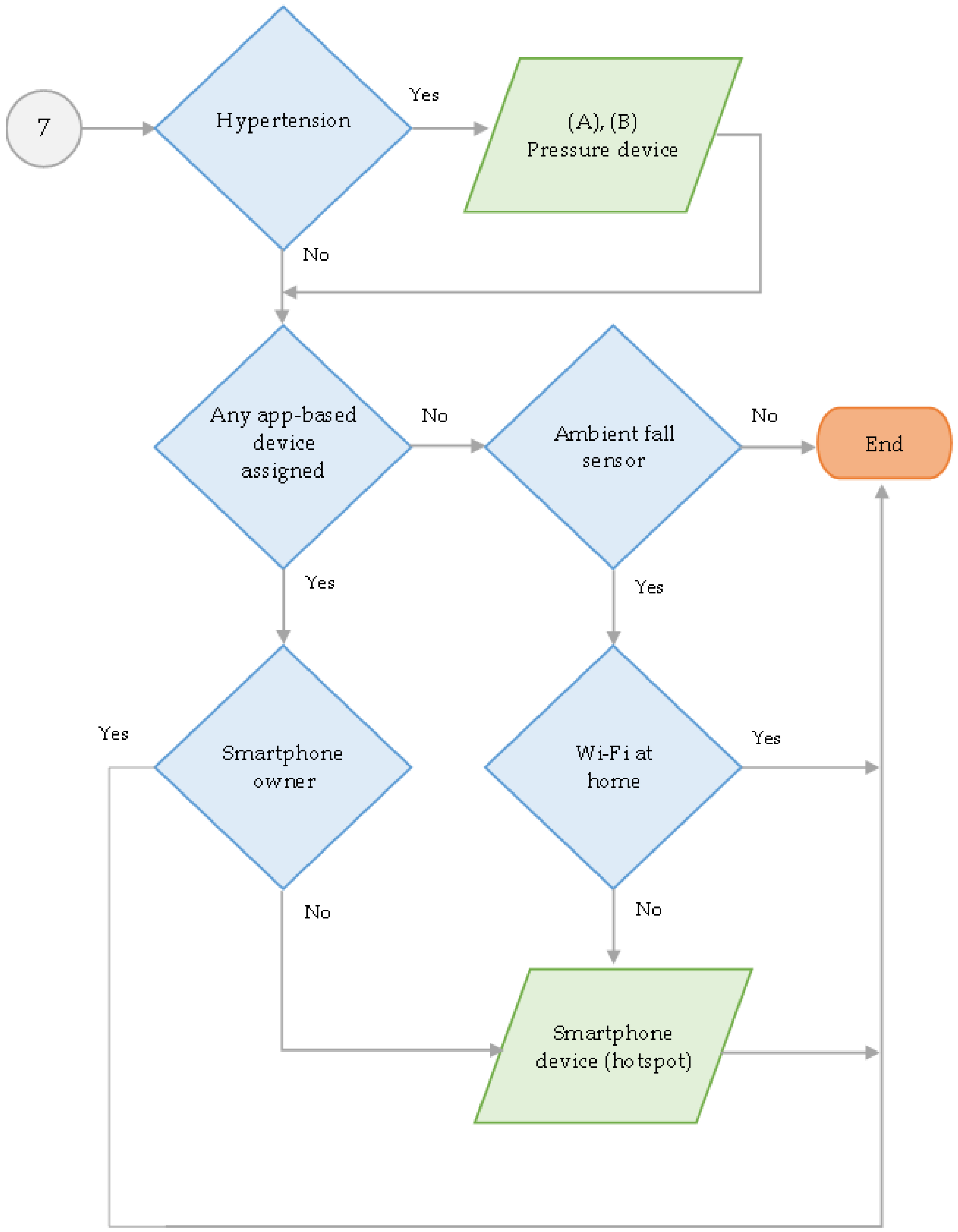
7
Hypertension
Yes
(A), (B)
Pressure device
No
Any app-based
device
assigned
No
Ambient fall
sensor
No
End
Yes
Yes
Yes
Smartphone
owner
Wi-Fi at
home
Yes
No
No
Smartphone
device (hotspot)
